# Identifying Risk Factors for Aspiration in Patients Hospitalized with Community-Acquired Pneumonia

**DOI:** 10.1155/2023/2198259

**Published:** 2023-07-18

**Authors:** Tianming Zhao, Yi Zhang, Kun Wang, Huan Yu, Lianjun Lin, Xueying Qin, Tao Wu, Dafang Chen, Yiqun Wu, Yonghua Hu

**Affiliations:** ^1^Department of Epidemiology and Biostatistics, School of Public Health, Peking University Health Science Center, Beijing 100191, China; ^2^Geriatric Department, Peking University First Hospital, Beijing 100034, China; ^3^Key Laboratory of Epidemiology of Major Diseases (Peking University), Ministry of Education, Beijing, China

## Abstract

**Background:**

Aspiration pneumonia (AP) is difficult to diagnose and has poor outcomes. This case-control study aimed to explore the risk factors and delineate the antibiotic usage for AP.

**Methods:**

Inpatients diagnosed with community-acquired pneumonia (CAP) from 2013 to 2017, enrolled in the urban employee basic medical insurance program in Beijing, were included and classified into the AP (*N* = 2,885) and non-AP (*N* = 53,825) groups. Risk factors were identified by logistic regression.

**Results:**

Older age (compared to 18–64 years, OR for 65–79 years: 4.3, 95% CI: 3.8–4.9; OR for >80 years: 6.3, 95% CI: 5.6–7.2), male (OR: 1.4, 95% CI: 1.3–1.5), cerebrovascular disease (OR: 3.1, 95% CI: 2.8–3.5), dementia (OR: 2.0, 95% CI: 1.8–2.1), vomiting (OR: 1.4, 95% CI: 1.2–1.7), Parkinson's disease (OR: 2.1, 95% CI: 1.8–2.4), and epilepsy (OR: 3.2, 95% CI: 2.8–3.7) were associated with an increased risk of AP. 92.8% of the AP patients received antibiotic therapy. Among them, patients treated with broad-spectrum antibiotics, antibiotics for injection, and combined antibiotics accounted for 93.3%, 97.9%, and 81.7%, respectively.

**Conclusions:**

Older age, male, and several comorbidities were independent risk factors for AP, and combined antibiotics treatments are common, which merits attention in accurate detection of AP in a high-risk population.

## 1. Introduction

Community-acquired pneumonia (CAP) is a dominant cause of hospitalization, death, and economic burden worldwide [1]. The prevalence of CAP is high among all age groups, especially for the elderly and patients with comorbidities [1–3]. Aspiration pneumonia (AP) is an infectious process caused by the inhalation of oropharyngeal secretions that are colonized by pathogenic bacteria [4]. It was reported that AP comprises 5%–24% of CAP cases [5]. Compared with other types of CAP, AP has a poorer outcome. A meta-analysis showed that the in-hospital and 30-day mortality of AP patients was significantly higher than in non-AP patients [6]. Besides, AP is difficult to diagnose. The generalized definition of AP is evidence of pneumonia with risk factors for aspiration, but silent aspirations often cause missed diagnosis [7]. Therefore, it is important to identify risk factors for AP and improve diagnosis and treatment for high-risk individuals. Several factors can increase the risk of AP, including age, male sex, smoking, dysphagia, reduced consciousness, neurologic disorders, gastroesophageal reflux, and tube feeding [7–10]. However, current studies of the risk factors for AP have limitations including a small sample size and varied diagnostic criteria.

AP is usually acute, with symptoms developing within hours to a few days [11]. In the past decades, there has been a shift in the pathogens involved in AP. In the 1970s, studies suggested that anaerobes are the predominant pathogens of AP [12]. In recent years, the prevalence of anaerobes significantly decreased, and *S. pneumoniae, H. influenzae, S. aureus,* and *Enterobacteriaceae* became the most common pathogens [7, 13, 14]. Based on the studies in the 1970s, antibiotics against anaerobes were the standard treatment for AP [4]. However, with the shift from anaerobes to more common CAP pathogens, treatment regimens altered consequently. The Infectious Disease Society of America (IDSA) and American Thoracic Society (ATS) guidelines recommend that anaerobic coverage is only indicated in the classic aspiration syndrome in patients with a history of decreased consciousness due to drugs, alcohol overdose, or seizures in patients with gingival disease or esophageal motility disorders [15]. However, several studies have reported inappropriate use of antianaerobic antibiotic coverage in clinical practice [9, 16], which can lead to the emergence of multidrug-resistant bacteria, adverse drug reactions, and higher costs [17]. Up to now, there has been a lack of large sample-sized studies delineating the empiric antibiotic therapy for AP in China. Thus, we designed this case-control study to explore the risk factors and delineate the antibiotic usage for AP.

## 2. Methods

### 2.1. Study Design and Database

This is a case-control study based on the Beijing Medical Claim Data for Employees (BMCDE). Patients with AP and CAP in the BMCDE database were included and classified as the case (AP) and control (non-AP) groups, respectively. The BMCDE database includes all the medical claim records for approximately 17.7 million working or retired employees enrolled in the urban employee basic medical insurance (UEBMI) in Beijing. The UEBMI is the main type of medical insurance in Beijing, covering more than 80% of all the residents [18]. The details of the BMCDE have been described previously [19]. Information collected in this study included demographic characteristics (age, sex, district, and occupation), dates of hospital admission, primary diagnosis, and medication use.

### 2.2. Participants

The AP patients were identified from the BMCDE database according to the following criteria: (1) inpatient cases from Jan 2013 to Dec 2017, (2) with a primary diagnosis of the ICD-10 code of J69.0 or diagnosed with medical terms of “aspiration pneumonia” [6, 20], and (3) with complete information on birth date and sex. As AP patients were identified only by the primary diagnosis, it can be assumed that these pneumonia patients were community-acquired [13]. The CAP patients were identified by similar criteria to AP, using the ICD-10 code of J10-J18 and the medical terms “pneumonia” [7, 15, 21]. The exclusion criteria were as follows: age <18 years and without age or sex. Eight patients were excluded because of incomplete information in this study.

### 2.3. Data Collection

Potential risk factors for aspiration include age, sex, dysphagia, cerebrovascular disease, head and neck cancer, gastroesophageal reflux, dementia, Parkinson's disease, hemiplegia, epilepsy, multiple sclerosis, lateral sclerosis, cardiac arrest, alcohol dependence, and sedative drug abuse (Supplementary methods) [2–5]. The Charlson Comorbidity Index of each patient was calculated according to their comorbidities (Supplementary methods) [22]. Antibacterial medications were abstracted by matching drug names with the Anatomical Therapeutic Chemical (ATC) classification system (World Health Organization Collaborating Centre for Drug Statistics Methodology) [23] to identify drug categories. Second-generation cephalosporins (J01DC), third-generation cephalosporins (J01DD), fourth-generation cephalosporins (J01DE), fluoroquinolones (J01MA), macrolides (J01FA, except erythromycin J01FA01), penicillins with extended-spectrum (J01CA), combinations of penicillins including beta-lactamase inhibitors (J01CR), carbapenems (J01DH), and streptomycins (J01GA) were classified as broad-spectrum antibiotics [19, 24, 25]. The other antibiotics were classified as narrow-spectrum antibiotics.

### 2.4. Statistical Analysis

Continuous variables were analyzed using Student's *t*-tests and expressed as means (SD). Categorical variables were compared using *χ*^2^ tests and reported as absolute frequencies and percentages. Logistic regression was used to identify the independent risk factors for AP. Odds ratios (ORs) and 95% confidence intervals (95% CIs) adjusted by age, sex, and Charlson Comorbidity Index were calculated. We stratified the patients into different groups by sex and age (18–64, 65–79, ≥80) and conducted subgroup analyses in each group. In the sensitivity analysis, data from 1 January 2016 to 31 December 2017 and data without readmissions within 30 days were analyzed using the logistic regression, respectively. A two-sided *P* value less than 0.05 was considered statistically significant. To delineate the antibiotic usage of the AP and non-AP groups, use frequencies and percentages of antibiotics, broad-spectrum antibiotics, antibiotics for injection, and combined antibiotics in different groups were presented. The first 10 most frequently prescribed antibiotics and their usage rates in each group were also reported. All analyses were performed using R version 4.1.0.

## 3. Results

### 3.1. Characteristics of Patients

A total of 56,710 patients were eligible for this study, with 2,885 patients in the AP group and 53,825 patients in the non-AP group. The mean age of patients was 68.0 (SD: 18.0) years, and 57.1% (32,387) of them were males. The characteristics of the patients are shown in Table 1. Compared with those in the non-AP group, patients in the AP group were older, more likely to be males, had higher Charlson index scores, and more likely to be comorbid with cerebrovascular disease, dementia, gastroesophageal reflux, vomit, Parkinson's disease, and epilepsy (Table 1).

### 3.2. Risk Factors Associated with AP

**Figure 1:**
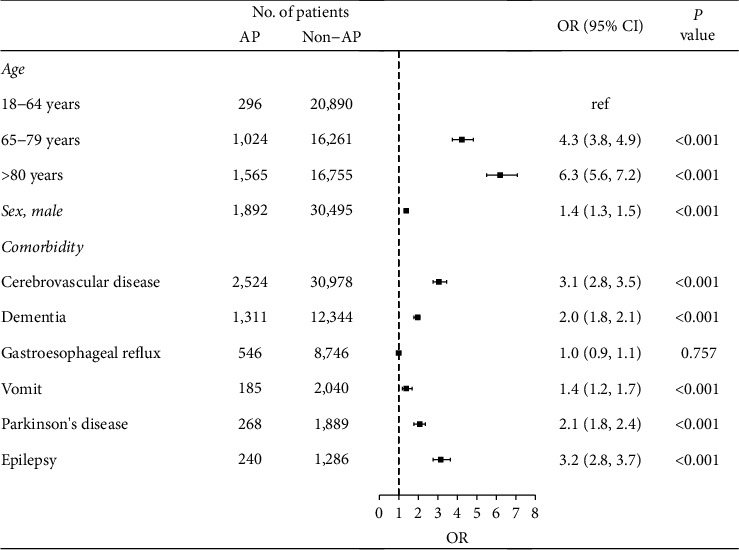
Risk factors for aspiration in patients hospitalized with CAP.

Older age, male sex, cerebrovascular disease, dementia, vomit, Parkinson's disease, and epilepsy were identified as independent risk factors for AP (Figure 1). The strengths of associations for risk factors and AP between sexes were similar for dementia, vomiting, and Parkinson's disease, stronger in males for cerebrovascular disease, and stronger in females for age and epilepsy (Table 2). The strengths of associations for risk factors and AP were stronger in those under 65 years (Table 3). The results were similar when only analyzing data from 1 January 2016 to 31 December 2017 (Supplementary Table 1–3) or data without readmissions within 30 days (Supplementary Table 4–6).

### 3.3. Prescription of Antibiotics

2,676 (92.8%) AP patients received antibiotic therapy. Among those treated with antibiotics, patients treated with broad-spectrum antibiotics, antibiotics for injection, and combined antibiotics accounted for 93.3%, 97.9%, and 81.7%, respectively. The percentage of antibiotic therapy was significantly higher in those aged ≥65 years (Supplementary Table 7). The top 10 most frequently prescribed antibiotics for AP patients were piperacillin (44.6%), meropenem (24.8%), levofloxacin (22.9%), moxifloxacin (20.2%), cefoperazone (17.9%), vancomycin (13.5%), imipenem (12.9%), cefixime (9.2%), latamoxef (9.1%), and etimicin (8.6%) (Table 4). The most frequently prescribed antibiotics were similar between sexes but lightly different between age groups (Supplementary Table 8).

As for non-AP patients, a total of 52,692 (97.9%) patients received antibiotic treatment. Patients treated with broad-spectrum antibiotics, antibiotics for injection, and combined antibiotics accounted for 96.6%, 98.9%, and 76.0%, respectively. The first 10 most frequently prescribed antibiotics were moxifloxacin (31.7%), levofloxacin (29.9%), piperacillin (26.3%), azithromycin (11.8%), meropenem (11.3%), cefdinir (10.3%), cefoperazone (10.3%), cefixime (8.7%), ceftazidime (8.4%), and imipenem (7.9%) (Table 4).

## 4. Discussion

Compared with common CAP, AP has a poorer outcome. Identifying risk factors for AP and improving diagnosis and treatment for high-risk individuals are critical. Based on a large sample of AP cases in the capital city of China, we identified older age, male sex, and comorbid with cerebrovascular disease, dementia, vomiting, Parkinson's disease, and epilepsy as independent risk factors for AP, and delineated the empiric antibiotic therapy for patients.

In the current study, increased age was a significant risk factor for AP. This result was consistent with some previous studies [26, 27]. Conversely, the study by Shariatzadeh et al. found that patients with AP were younger than those with nonaspiration CAP [5]. The study included 1,946 inpatients diagnosed with pneumonia from 2001 to 2002 in Alberta, Canada. They adopted age as a continuous variable instead of a categorical variable and used Student's *t* -test to compare the difference in the mean age between patients with community-acquired aspiration pneumonia and patients with community-acquired nonaspiration pneumonia. Differences in characteristics of the study population, diagnostic criteria, and statistical technique may cause the conflicting results on association between age and aspiration. Based on studies on the effects of aging on swallowing and cough reflex, it is suggested that aging alone does not increase the risk of AP [28]. It is found that older people swallow more slowly, but safety is not comprised [29–32]. Cough reflex does not decrease with advancing age [33]. But age is associated with an increasing incidence of neurological disorders, which impair swallow function and cough reflex and thus increase the risk of aspiration [28]. In our results, we observed a significantly higher mean age in AP patients, as well as significantly higher risks of AP in the elder groups even after adjusting for several comorbidities. Considering the increasing old population and the compromised immunity and physical degeneration in the elderly, the care for the elderly with comorbidities associated with AP should be strengthened. In accordance with previous studies [9, 26, 27, 34], we found that males have a higher risk than females (OR, 1.4; 95% CI, 1.3–1.5), which suggests that we should pay special attention to males in clinical diagnosis and prevention of AP.

We identified neurological disorders including cerebrovascular disease, dementia, Parkinson's disease, and epilepsy as independent risk factors, which are similar to those in multiple studies [8–10, 27]. Neurological disorders can cause swallowing difficulty, impaired cough reflex, and reduced consciousness, which increase the chance that aspirated material will reach the lung. Previous studies suggested that dysphagia is an established risk factor for AP [7, 11, 28]. However, the incidence of dysphagia in our data was too low to give an accurate statistical result. It is possible that dysphagia tends to be regarded as a symptom rather than a disease, which led to the low diagnostic rate of dysphagia in our data. This emphasizes the importance of diagnosing dysphagia in CAP patients.

Several studies indicated that gastroesophageal reflux and vomiting are risk factors for AP [7, 8, 35]. In these situations, gastric contents containing pathogens and gastric acid reflux into the esophagus and larynx, even into the lung when the defense mechanism against aspiration is impaired, can injure the respiratory epithelial tissue and cause bacterial infection. In line with previous studies, our study demonstrated vomiting has a significant association with AP. However, gastroesophageal reflux showed a contrary result. One possible reason is that gastroesophageal reflux can cause more silent aspirations during sleep which are hard to observe, so gastroesophageal reflux-induced AP is more likely to be misdiagnosed as typical CAP.

In the subgroup analysis by sex, we found that the OR of cerebrovascular disease in the male group is higher than that in the female group, whereas the OR of epilepsy in the male group is lower than that in the female group. The results are consistent with sensitivity analysis. This may be due to the differences in biological (e.g., sex hormone) and behavioral (e.g., tobacco use and alcohol drinking) characteristics between sexes. Since female sex hormone can influence neuronal excitability, seizure frequency of females with epilepsy increases during specific phases of the menstrual cycle, known as catamenial epilepsy [36], which may lead to a higher risk for AP in females with epilepsy. In the subgroup analysis by age, the associations between cerebrovascular disease, dementia, and epilepsy with AP in the younger group were higher, which indicated a possible higher misdiagnosed silent aspiration in the elderly. The results again emphasize the importance of the care for the elderly with pneumonia.

According to the results, the rate of antibiotic therapy in both groups exceeded 90%. The non-AP group had a slightly higher antibiotic usage rate than the AP group. The rate of combined antibiotics in the AP group was higher than in the non-AP group. The result was similar to that of a previous study [37]. Antibiotic therapy is associated with the microbiology of AP patients. A study on microbiology of hospital- and community-acquired aspiration pneumonia found that Gram-negative bacilli were the predominant cultured microorganism and that the main isolated bacteria demonstrated high and multiantibiotic resistance [38]. Wang et al. found that the mixed infection rate was higher in AP patients, and of those main infection pathogenic bacteria, the *Acinetobacter baumannii* infection rate was also higher than in non-AP patients [37]. However, the use of potent antibiotics may cause the occurrence of multidrug-resistant bacteria, which increases the difficulty to treat AP.

The novelty of this study lies in that it is a large sample-sized study and that we compared the risk of comorbidities in different populations which promote a more targeted diagnosis of aspiration pneumonia. This study has limitations. First, the geographic area of this study was limited to Beijing. China has a vast territory and significant urban-rural differences. Given the potential differences in characteristics between populations settled in Beijing and other parts, the results should be carefully adapted to other areas in China. Second, due to limited data, other potential risk factors such as tube feeding, smoking, and the use of proton-pump inhibitors or H2-blocker for gastric acid suppression could not be analyzed in the study. The lack of these covariates may cause errors in the results. Third, the results of the bacterial culture and the drug sensitivity test are inaccessible, so we could not judge the rationality of clinical antibiotic treatment or compare the antibiotic resistance difference between the groups. Fourth, due to data availability, we have no access to the outcomes or prognosis of the study population. Comparing the outcomes and prognosis between the groups is valuable for assessing the impact of the identified risk factors. We could not analyze the impact of risk factors on outcomes of AP patients for the lack of this information. Based on our limitations, future studies on aspiration pneumonia can make following improvements. Data in different areas could be combined to improve the representativeness of the study population. The clinical data should include more items such as outcomes and prognosis, laboratory findings, and other potential risk factors to permit a comprehensive assessment of the risk factors for aspiration and rationality of clinical antibiotic usage.

In conclusion, older age, male, and several comorbidities were associated with increased risks of aspiration pneumonia. Combined antibiotic treatments are common in AP patients. The results merit attention in the accurate detection of AP in the elderly and high-risk population.

## Figures and Tables

**Table 1:** Characteristics of patients.

	Total (*N* = 56,710)	AP (*N* = 2,885)	Non-AP (*N* = 53,825)	*P* value
Age, mean (SD), yrs	68.0 (18.0)	79.1 (9.6)	67.4 (18.2)	<0.001
Age groups				<0.001
18–64	21,105 (37.2)	296 (10.3)	20,890 (38.7)	
65–79	17,285 (30.5)	1,024 (35.5)	16,261 (30.2)	
≥80	18,320 (32.3)	1,565 (54.2)	16,755 (31.1)	
Males	32,387 (57.1)	1,892 (65.6)	30,495 (56.7)	<0.001
Charlson Index, mean (SD)	3.3 (2.9)	4.2 (2.6)	3.2 (2.9)	<0.001
Comorbidities				
Cerebrovascular disease	33,502 (59.1)	2,524 (87.5)	30,978 (57.6)	<0.001
Dementia	13,655 (24.1)	1,311 (45.4)	12,344 (22.9)	<0.001
Gastroesophageal reflux	9,292 (16.4)	546 (18.9)	8,746 (16.3)	<0.001
Vomiting	2,225 (3.9)	185 (6.4)	2,040 (3.8)	<0.001
Parkinson	2,157 (3.8)	268 (9.3)	1,889 (3.5)	<0.001
Epilepsy	1,526 (2.7)	240 (8.3)	1,286 (2.4)	<0.001

**Table 2:** Risk factors for aspiration in patients hospitalized with CAP by different sexes.

	Male		Female	
	OR (95% CI)	*P* value	OR (95% CI)	*P* value
Age groups				
18–64	Ref		Ref	
65–79	3.3 (2.9, 3.9)	<0.001	9.0 (6.7, 12.2)	<0.001
≥80	4.5 (3.9, 5.2)	<0.001	15.4 (11.6, 20.8)	<0.001
Comorbidities				
Cerebrovascular disease	4.0 (3.4, 4.6)	<0.001	2.0 (1.7, 2.5)	<0.001
Dementia	2.0 (1.8, 2.2)	<0.001	2.0 (1.7, 2.3)	<0.001
Gastroesophageal reflux	1.1 (0.9, 1.2)	0.365	0.9 (0.7, 1.0)	0.118
Vomiting	1.6 (1.3, 2.0)	<0.001	1.3 (1.0, 1.6)	0.074
Parkinson	2.1 (1.8, 2.5)	<0.001	2.0 (1.6, 2.5)	<0.001
Epilepsy	2.8 (2.3, 3.3)	<0.001	4.4 (3.4, 5.6)	<0.001

**Table 3:** Risk factors for aspiration in patients hospitalized with CAP by different ages.

	18–64 years		65–79 years		≥80 years	
	OR (95% CI)	*P* value	OR (95% CI)	*P* value	OR (95% CI)	*P* value
Male	4.0 (3.0, 5.5)	<0.001	1.5 (1.3, 1.7)	<0.001	1.2 (1.0, 1.3)	0.008
Comorbidities						
Cerebrovascular disease	9.1 (7.0, 11.9)	<0.001	3.1 (2.5, 3.9)	<0.001	1.8 (1.6, 2.2)	<0.001
Dementia	5.5 (4.2, 7.0)	<0.001	2.3 (2.0, 2.6)	<0.001	1.5 (1.4, 1.7)	<0.001
Gastroesophageal reflux	1.7 (1.2, 2.4)	0.003	1.1 (1.0, 1.3)	0.116	0.8 (0.7, 0.9)	<0.001
Vomiting	2.6 (1.3, 4.4)	0.002	1.5 (1.1, 1.9)	0.004	1.3 (1.1, 1.6)	0.011
Parkinson	4.9 (2.5, 8.5)	0.013	2.0 (1.6, 2.4)	<0.001	1.9 (1.6, 2.3)	<0.001
Epilepsy	13.2 (9.3, 18.3)	<0.001	3.7 (3.0, 4.6)	<0.001	1.6 (1.2, 2.1)	<0.001

**Table 4:** Antibiotic treatment in AP and non-AP patients.

	AP	Non-AP
Percentage of antibiotics usage, *N* (%)		
Overall antibiotics	2,676 (92.8)	52,692 (97.9)
Broad-spectrum antibiotics	2,625 (98.1)	51,972 (98.6)
Antibiotics for injection	2,619 (97.9)	52,093 (98.9)
Combined antibiotics	2,187 (81.7)	40,047 (76.0)
The top ten antibiotics (%)		
1	Piperacillin (44.6)	Moxifloxacin (31.7)
2	Meropenem (24.8)	Levofloxacin (29.9)
3	Levofloxacin (22.9)	Piperacillin (26.3)
4	Moxifloxacin (20.2)	Azithromycin (11.8)
5	Cefoperazone (17.9)	Meropenem (11.3)
6	Vancomycin (13.5)	Cefdinir (10.3)
7	Imipenem (12.9)	Cefoperazone (10.3)
8	Cefixime (9.2)	Cefixime (8.7)
9	Latamoxef (9.1)	Ceftazidime (8.4)
10	Etimicin (8.6)	Imipenem (7.9)

## Data Availability

The data that support the findings of this study are available from the corresponding authors upon reasonable request.

## Supplementary Materials

Supplementary Materials The supplementary file provides our detailed method to identify the risk factors for aspiration and the Charlson comorbidities. Supplementary Tables 1–3: the results of data from 2016 to 2017. Supplementary Tables 4–6: the results of data without readmissions within 30 days. Supplementary Table 7: the percentage of antibiotic usage for AP patients. Supplementary Table 8: the top ten most frequently used antibiotics for AP patients.
