# Supplementary material for: Identifying Risk Factors for Aspiration in Patients Hospitalized with Community-Acquired Pneumonia
Source: Int J Clin Pract. 2023 Jul 18;2023:2198259. doi: 10.1155/2023/2198259 (PMC10368512; doi:10.1155/2023/2198259)
Supplement: Supplementary Materials — The supplementary file provides our detailed method to identify the risk factors for aspiration and the Charlson comorbidities. Supplementary Tables 1–3: the results of data from 2016 to 2017. Supplementary Tables 4–6: the results of data without readmissions within 30 days. Supplementary Table 7: the percentage of antibiotic usage for AP patients. Supplementary Table 8: the top ten most frequently used antibiotics for AP patients. [file 2198259.f1.docx]

**Identifying risk factors for aspiration in patients hospitalized with community-acquired pneumonia**

**Supplementary file**

Tianming Zhao 1, Yi Zhang 1, Kun Wang 1, Huan Yu 1, Lianjun Lin 2, Xueying Qin 1, Tao Wu 1, Dafang Chen 1, Yiqun Wu 1*, Yonghua Hu 1*

* Corresponding authors.

**Affiliation:**

1 Department of Epidemiology and Biostatistics, School of Public Health, Peking University Health Science Center, 100191

2 Geriatric Department, Peking University First Hospital, 100034

**Corresponding to:**

Yiqun Wu, Ph.D., Department of Epidemiology and Biostatistics, School of Public Health, Peking University, No. 38 Xueyuan Road, 100191 Beijing, China, Tel: +86-138-1049-0718, Fax: +86-10-82801528, E-mail: qywu118@163.com

Yonghua Hu, MD, Department of Epidemiology and Biostatistics, School of Public Health, Peking University, No. 38 Xueyuan Road, 100191 Beijing, China, Tel: +86-135-0136-1139, Fax: +86-10-82801189, E-mail: [yhhu@bjmu.edu.cn](mailto:yhhu@bjmu.edu.cn)

**Methods**

**Identification of risk factors for aspiration**

Risk factors for aspiration included gastroesophageal reflux disease, vomiting, head and neck cancer, dysphagia, cerebrovascular disease, Parkinson’s disease, dementia, impaired level of consciousness (convulsions, epilepsy, cardiac arrest, intoxication, alcoholism, sedative-hypnotic drug overdose), multiple sclerosis, lateral sclerosis of the spinal cord [1-4]. ICD-10 code, as well as diagnosis terms, were used to identify the diseases. The Chinese medical terms of one disease may take many forms. The Chinese medical terms of the risk factors for aspiration were obtained by first searching the ICD-10 codes to obtain the corresponding comorbidity cases and their Chinese medical terms. Then, 1000 Chinese textual diagnoses for each ICD-10 code were reviewed to obtain the common textual diagnosis fields. ICD-10 codes are listed below.

Gastroesophageal reflux: ICD-10 code of K21.0, K21.9 [5, 6]

Head and neck cancer: ICD-10 code of C0, C10, C11, C12, C13, C14, C30, C31, C32, C73 [7-9]

Vomiting: ICD-10 code of R11 [10]

Dysphagia: ICD-10 code of R13 [11]

Cerebrovascular disease: ICD-10 code of I60, I61, I62, I63, I64, I65, I66, I67, I68, I69, G45 [12]

Multiple sclerosis: ICD-10 code of G35 [13]

Parkinson’s: ICD-10 code of G20, G21, G22 [14]

Dementia: ICD-10 code of G31.0, G31.82, G23.1, F00, F01, F02, F03, F04, F05, I69, G45 [15]

Lateral sclerosis: ICD-10 code of G12.2 [16]

Epilepsy and seizures: ICD-10 code of G40, G41 [17]

Convulsions: ICD-10 code of R56

Cardiac arrest: ICD-10 code of I46

Alcohol dependence: ICD-10 code of F10, Y90, Y91 [18]

Sedative drug abuse: ICD-10 code of F13, T41, T42 [19]

**Identification of** **the Charlson comorbidities**

Charlson comorbidities were identified by both the ICD-10 code and diagnosed medical terms. ICD-10 codes for the Charlson comorbidities were obtained from references [20]. The Chinese medical terms of one disease may take many forms. The Chinese medical terms of the Charlson comorbidities were obtained by first searching the ICD-10 codes to obtain the corresponding comorbidity cases and their medical terms. Then, 1000 Chinese textual diagnoses for each ICD-10 code were reviewed to obtain the common textual diagnosis fields. ICD-10 is consistent with the reference[20].

**References**

1. Taylor, J.K., et al., *Risk factors for aspiration in community-acquired pneumonia: analysis of a hospitalized UK cohort.* Am J Med, 2013. **126**(11): p. 995-1001.

2. Mandell, L.A. and M.S. Niederman, *Aspiration Pneumonia.* N Engl J Med, 2019. **380**(7): p. 651-663.

3. Khan, A., R. Carmona, and M. Traube, *Dysphagia in the elderly.* Clin Geriatr Med, 2014. **30**(1): p. 43-53.

4. Lee, A.S. and J.H. Ryu, *Aspiration Pneumonia and Related Syndromes.* Mayo Clin Proc, 2018. **93**(6): p. 752-762.

5. Dahlen, H.G., et al., *Gastro-oesophageal reflux: a mixed methods study of infants admitted to hospital in the first 12 months following birth in NSW (2000-2011).* BMC Pediatr, 2018. **18**(1): p. 30.

6. Uno, S., et al., *Comorbidities associated with nontuberculous mycobacterial disease in Japanese adults: a claims-data analysis.* BMC Pulm Med, 2020. **20**(1): p. 262.

7. Bøje, C.R., et al., *The impact of comorbidity on outcome in 12 623 Danish head and neck cancer patients: a population based study from the DAHANCA database.* Acta Oncol, 2013. **52**(2): p. 285-93.

8. Nieminen, M., et al., *Challenges in diagnosing head and neck cancer in primary health care.* Ann Med, 2021. **53**(1): p. 26-33.

9. Overgaard, J., et al., *The Danish Head and Neck Cancer database.* Clin Epidemiol, 2016. **8**: p. 491-496.

10. Benary, D., et al., *Ondansetron Prescription Is Associated With Reduced Return Visits to the Pediatric Emergency Department for Children With Gastroenteritis.* Ann Emerg Med, 2020. **76**(5): p. 625-634.

11. Krasnodębska, P., et al., *Diagnosis in Muscle Tension Dysphagia.* Otolaryngol Pol, 2020. **75**(1): p. 16-22.

12. Ekker, M.S., et al., *Association of Stroke Among Adults Aged 18 to 49 Years With Long-term Mortality.* Jama, 2019. **321**(21): p. 2113-2123.

13. Taylor, T.R., et al., *Prevalence and demographics of multiple sclerosis-associated uveitis: a UK biobank study.* Mult Scler Relat Disord, 2020. **43**: p. 102209.

14. Pitcher, T.L., et al., *Parkinson's disease across ethnicities: A nationwide study in New Zealand.* Mov Disord, 2018. **33**(9): p. 1440-1448.

15. Moura, L., et al., *Identifying Medicare beneficiaries with dementia.* J Am Geriatr Soc, 2021. **69**(8): p. 2240-2251.

16. Larson, T.C., et al., *Amyotrophic Lateral Sclerosis Mortality in the United States, 2011-2014.* Neuroepidemiology, 2018. **51**(1-2): p. 96-103.

17. Bergen, D.C., E. Beghi, and M.T. Medina, *Revising the ICD-10 codes for epilepsy and seizures.* Epilepsia, 2012. **53 Suppl 2**: p. 3-5.

18. Langley, J., et al., *Use of alcohol intoxication codes for serious non-fatal hospitalised injury.* Injury, 2013. **44**(11): p. 1472-6.

19. Tyndall Snow, L.M., et al., *Descriptive exploration of overdose codes in hospital and emergency department discharge data to inform development of drug overdose morbidity surveillance indicator definitions in ICD-10-CM.* Inj Prev, 2021. **27**(S1): p. i27-i34.

20. Thygesen, S.K., et al., *The predictive value of ICD-10 diagnostic coding used to assess Charlson comorbidity index conditions in the population-based Danish National Registry of Patients.* BMC Med Res Methodol, 2011. **11**: p. 83.

**Supplementary Table 1 Risk factors for aspiration in patients hospitalized with community-acquired pneumonia (2016-2017)**

|  | No. of AP | No. of non-AP | OR (95% CI) | *P*-value |
| --- | --- | --- | --- | --- |
| Age group |  |  |  |  |
| 18-64 | 117 | 10,565 | ref |  |
| 65-79 | 399 | 7,971 | 4.4 (3.6, 5.5) | <0.001 |
| ≥80 | 816 | 9,712 | 7.4 (6.1, 9.2) | <0.001 |
| Sex. Male | 847 | 15,871 | 1.3 (1.2, 1.5) | <0.001 |
| District. City | 1,127 | 22,160 | 1.1 (1.0, 1.3) | 0.092 |
| Comorbidity |  |  |  |  |
| Cerebrovascular disease | 1,144 | 16,094 | 2.6 (2.2, 3.1) | <0.001 |
| Dementia | 566 | 6,355 | 1.8 (1.6, 2.0) | <0.001 |
| Gastroesophageal reflux | 241 | 4,481 | 0.9 (0.8, 1.1) | 0.488 |
| Vomit | 64 | 1,007 | 1.1 (0.9, 1.4) | 0.391 |
| Parkinson | 104 | 935 | 1.8 (1.5, 2.2) | <0.001 |
| Epilepsy | 89 | 606 | 2.9 (2.2, 3.6) | <0.001 |

**Supplementary Table 2 Risk factors for aspiration in patients hospitalized with CAP, by different sexes (2016-2017)**

|  | Male | |  | Female | |
| --- | --- | --- | --- | --- | --- |
|  | OR (95% CI) | *P*-value |  | OR (95% CI) | *P*-value |
| Age group |  |  |  |  |  |
| 18-64 | ref |  |  | ref |  |
| 65-79 | 3.3 (2.9, 3.9) | <0.001 |  | 9.0 (6.7, 12.2) | <0.001 |
| ≥80 | 4.5 (3.9, 5.2) | <0.001 |  | 15.4 (11.6, 20.8) | <0.001 |
| Comorbidity |  |  |  |  |  |
| Cerebrovascular disease | 4.0 (3.4, 4.6) | <0.001 |  | 2.0 (1.7, 2.5) | <0.001 |
| Dementia | 2.0 (1.8, 2.2) | <0.001 |  | 2.0 (1.7, 2.3) | <0.001 |
| Gastroesophageal reflux | 1.1 (0.9, 1.2) | 0.365 |  | 0.9 (0.7, 1.0) | 0.118 |
| Vomit | 1.6 (1.3, 2.0) | <0.001 |  | 1.3 (1.0, 1.6) | 0.074 |
| Parkinson | 2.1 (1.8, 2.5) | <0.001 |  | 2.0 (1.6, 2.5) | <0.001 |
| epilepsy | 2.8 (2.3, 3.3) | <0.001 |  | 4.4 (3.4, 5.6) | <0.001 |

**Supplementary Table 3 Risk factors for aspiration in patients hospitalized with CAP, by different ages (2016-2017)**

|  | 18-64 years | |  | 65-79 years | |  | ≥80 years | |
| --- | --- | --- | --- | --- | --- | --- | --- | --- |
|  | OR (95% CI) | *P*-value |  | OR (95% CI) | *P*-value |  | OR (95% CI) | *P*-value |
| Male | 3.0 (2.0, 4.7) | <0.001 |  | 1.7 (1.3, 2.1) | <0.001 |  | 1.1 (0.9, 1.3) | 0.290 |
| Comorbidity |  |  |  |  |  |  |  |  |
| Cerebrovascular disease | 6.1 (4.2, 9.1) | <0.001 |  | 3.1 (2.3, 4.3) | <0.001 |  | 1.7 (1.4, 2.2) | <0.001 |
| Dementia | 5.7 (3.8, 8.4) | <0.001 |  | 2.6 (2.1, 3.2) | <0.001 |  | 1.3 (1.1, 1.5) | <0.001 |
| Gastroesophageal reflux | 1.5 (0.8, 2.6) | 0.138 |  | 1.4 (1.1, 1.7) | 0.011 |  | 0.7 (0.6, 0.9) | 0.002 |
| Vomit | 0.5 (0.0, 2.4) | 0.523 |  | 1.4 (0.8, 2.1) | 0.195 |  | 1.1 (0.8, 1.5) | 0.614 |
| Parkinson | 3.2 (0.8, 8.8) | 0.051 |  | 2.7 (1.9, 3.6) | <0.001 |  | 1.4 (1.0, 1.8) | 0.024 |
| epilepsy | 9.8 (5.2, 17.1) | <0.001 |  | 4.8 (3.4, 6.7) | <0.001 |  | 1.3 (0.8, 1.9) | 0.276 |

**Supplementary Table 4 Risk factors for aspiration in patients hospitalized with community-acquired pneumonia (without readmissions within 30 days)**

|  | No. of AP | No. of non-AP | OR (95% CI) | *P*-value |
| --- | --- | --- | --- | --- |
| Age group |  |  |  |  |
| 18-64 | 277 | 20553 | ref |  |
| 65-79 | 969 | 15784 | 4.4 (3.9, 5.1) | <0.001 |
| ≥80 | 1479 | 16042 | 6.6 (5.8, 7.5) | <0.001 |
| Sex. Male | 1798 | 29566 | 1.5 (1.3, 1.6) | <0.001 |
| District. City | 2306 | 41202 | 1.2 (1.0, 1.3) | 0.006 |
| Comorbidity |  |  |  |  |
| Cerebrovascular disease | 2392 | 29937 | 3.2 (2.8, 3.7) | <0.001 |
| Dementia | 1238 | 11858 | 2.0 (1.8, 2.2) | <0.001 |
| Gastroesophageal reflux | 515 | 8503 | 1.0 (0.9, 1.1) | 0.652 |
| Vomit | 177 | 1982 | 1.5 (1.2, 1.7) | <0.001 |
| Parkinson | 254 | 1783 | 2.1 (1.8, 2.4) | <0.001 |
| Epilepsy | 226 | 1230 | 3.2 (2.8, 3.8) | <0.001 |

**Supplementary Table 5 Risk factors for aspiration in patients hospitalized with CAP, by different sexes (without readmissions within 30 days)**

|  | Male | |  | Female | | |
| --- | --- | --- | --- | --- | --- | --- |
|  | OR (95% CI) | *P*-value |  | OR (95% CI) | *P*-value | |
| Age group |  |  |  |  |  | |
| 18-64 | ref |  |  | ref |  | |
| 65-79 | 3.4 (2.9, 4.0) | <0.001 |  | 9.9 (7.3, 13.8) | <0.001 | |
| ≥80 | 4.6 (4.0, 5.3) | <0.001 |  | 17.6 (13.0, 24.3) | <0.001 | |
| Comorbidity |  |  |  |  |  | |
| Cerebrovascular disease | 4.1 (3.5, 4.8) | <0.001 |  | 2.1 (1.7, 2.6) | <0.001 | |
| Dementia | 2.0 (1.8, 2.2) | <0.001 |  | 2.0 (1.8, 2.3) | <0.001 |  |
| Gastroesophageal reflux | 1.1 (0.9, 1.2) | 0.243 |  | 0.8 (0.7, 1.0) | 0.033 |  |
| Vomit | 1.6 (1.3, 2.0) | <0.001 |  | 1.3 (1.0, 1.6) | 0.065 |  |
| Parkinson | 2.2 (1.8, 2.6) | <0.001 |  | 2.0 (1.6, 2.6) | <0.001 |  |
| epilepsy | 2.9 (2.4, 3.4) | <0.001 |  | 4.3 (3.3, 5.5) | <0.001 |  |

**Supplementary Table 6 Risk factors for aspiration in patients hospitalized with CAP, by different ages (without readmissions within 30 days)**

|  | 18-64 years | |  | 65-79 years | |  | ≥80 years | |
| --- | --- | --- | --- | --- | --- | --- | --- | --- |
|  | OR (95% CI) | *P*-value |  | OR (95% CI) | *P*-value |  | OR (95% CI) | *P*-value |
| Male | 4.5 (3.3, 6.3) | <0.001 |  | 1.6 (1.4, 1.8) | <0.001 |  | 1.2 (1.0, 1.3) | 0.006 |
| Comorbidity |  |  |  |  |  |  |  |  |
| Cerebrovascular disease | 10.2 (7.7, 13.6) | <0.001 |  | 3.2 (2.6, 4.0) | <0.001 |  | 1.9 (1.6, 2.2) | <0.001 |
| Dementia | 5.9 (4.5, 7.6) | <0.001 |  | 2.4 (2.1, 2.7) | <0.001 |  | 1.5 (1.4, 1.7) | <0.001 |
| Gastroesophageal reflux | 1.5 (1.0, 2.1) | 0.046 |  | 1.3 (1.1, 1.5) | 0.003 |  | 0.8 (0.7, 0.9) | <0.001 |
| Vomit | 2.8 (1.5, 4.9) | <0.001 |  | 1.7 (1.3, 2.2) | <0.001 |  | 1.3 (1.0, 1.6) | 0.019 |
| Parkinson | 5.5 (2.9, 9.7) | <0.001 |  | 2.2 (1.8, 2.7) | <0.001 |  | 1.9 (1.6, 2.3) | <0.001 |
| epilepsy | 14.0 (9.9, 19.6) | <0.001 |  | 4.0 (3.2, 4.9) | <0.001 |  | 1.6 (1.2, 2.0) | <0.001 |

**Supplementary Table 7 The percentage of antibiotic usage for AP patients**

|  | Overall | Sex | |  | Age | | |
| --- | --- | --- | --- | --- | --- | --- | --- |
|  |  | Male | Female |  | 18-64 | 65-79 | ≥80 |
| Antibiotics | 2,676 (92.8) | 1,782 (94.2) | 894 (90.0) |  | 257 (86.8) | 972 (94.9) | 1,447 (92.5) |
| Broad-spectrum antibiotics | 2,625 (98.1) | 1,745 (97.9) | 880 (98.4) |  | 248 (96.5) | 958 (98.6) | 1,419 (98.1) |
| Antibiotics for injection | 2,619 (97.9) | 1,745 (97.9) | 874 (97.8) |  | 247 (96.1) | 954 (98.1) | 1,418 (98.0) |
| Combined antibiotics | 2,187 (81.7) | 1,468 (80.4) | 719 (80.4) |  | 201 (78.2) | 811 (83.4) | 1,175 (81.2) |

**Supplementary Table 8 The top ten most frequently used antibiotics for AP patients**

| Rank | Overall | Sex | |  | Age | | |
| --- | --- | --- | --- | --- | --- | --- | --- |
|  |  | Male | Female |  | 18-64 | 65-79 | ≥80 |
| 1 | J01CR05 (44.6) | J01CR05 (45.8) | J01CR05 (42.2) |  | J01CR05 (37.5) | J01CR05 (44.8) | J01CR05 (45.8) |
| 2 | J01DH02 (24.8) | J01DH02 (26.4) | J01DH02 (21.9) |  | J01MA12 (22.3) | J01DH02 (24.2) | J01DH02 (26.3) |
| 3 | J01MA12 (22.9) | J01MA12 (24.5) | J01MA12 (19.9) |  | J01MA14 (20.3) | J01MA12 (23.1) | J01MA12 (22.9) |
| 4 | J01MA14 (20.2) | J01MA14 (20.8) | J01MA14 (19.1) |  | J01DH02 (19.3) | J01MA14 (22.1) | J01MA14 (19.0) |
| 5 | J01DD62 (17.9) | J01DD62 (20.1) | J01DD62 (13.5) |  | J01DD62 (17.9) | J01DD62 (21.2) | J01DD62 (15.7) |
| 6 | J01XA01 (13.5) | J01XA01 (14.1) | J01XA01 (12.3) |  | J01DH51 (12.2) | J01XA01 (15.9) | J01XA01 (12.6) |
| 7 | J01DH51 (12.9) | J01DH51 (13.8) | J01DH51 (11.1) |  | J01GB13 (11.1) | J01DH51 (14.3) | J01DH51 (12.1) |
| 8 | J01DD08 (9.2) | J01GB13 (9.9) | J01DD06 (9.7) |  | J01DD15 (10.1) | J01DD08 (10.6) | J01DD06 (9.8) |
| 9 | J01DD06 (9.1) | J01DD08 (9.4) | J01DD08 (9.0) |  | J01XA01 (9.8) | J01GB13 (10.0) | J01DD08 (8.9) |
| 10 | J01GB13 (8.6) | J01DD06 (8.8) | J01DD52 (8.7) |  | J01DD02 (8.4) | J01DD52 (8.9) | J01XD03 (8.8) |

J01CR05: Piperacillin; J01DH02: Meropenem; J01MA12: Levofloxacin; J01MA14: Moxifloxacin; J01DD62: Cefoperazone; J01XA01: Vancomycin; J01DH51: Imipenem; J01DD08: Cefixime; J01DD06: Latamoxef; J01GB13: Etimicin; J01DD52: Cefotaxime; J01DD15: Cefdinir; J01DD02: Ceftazidime; J01XD03: Ornidazole
